# A New Technique Based on Voronoi Tessellation to Assess the Space-Dependence of Categorical Variables

**DOI:** 10.3390/e21080774

**Published:** 2019-08-08

**Authors:** Pedro J. Zufiria, Miguel Á. Hernández-Medina

**Affiliations:** ETS Ingenieros de Telecomunicación, Information Processing and Telecommunications Center (IPTC), Universidad Politécnica de Madrid, 28040 Madrid, Spain

**Keywords:** spatial correlation, independence indices, Voronoi tessellation, entropy

## Abstract

Based on a sample of geolocated elements, each of them labeled with a (not necessarily ordered) categorical feature, several indexes for assessing the relationship between the geolocation variables (latitude and longitude) and the categorical variable are evaluated. Among these indexes, a new one based on a Voronoi tessellation presents several advantages since it does not require a variable transformation or a previous discretization; in addition, simulations show that this index is considerably robust when compared with the previously known ones. Finally, the use of the presented indexes is also illustrated by analyzing the geolocation of communities in some communication networks derived from Call Detail Records.

## 1. Introduction

The statistical analysis of space-related information is a long-developed research field with applications in biology [1], geology [2,3], sociology [4], etc., where different measures have been defined for assessing different features such as spatial dispersion [5,6], spatial autocorrelation [7,8], or spatial homogeneity [9]. Presently, geolocated information is becoming widely available from many sources of data such as Call Detail Records (CDRs) of telephone operators [10,11], vehicle geolocation systems [12,13], Internet of Things (IOT) architectures [14,15] or population surveys [16]. In general, these sets of geolocated data gather sample vectors which contain at least two continuous variables determining the latitude and longitude of the actor, and some other categorical variables which may take values belonging to a (not necessarily ordered) finite set of labels. In addition, new scenarios where these types of data are encountered are becoming common when modelling social networks. In this context, for instance, secondary variables are frequently computed for labeling network communities [17], whose relationships with geolocation have been recently analyzed for different countries at global and city scopes [18].

The above-mentioned scenarios encourage the study and development of new tools for the statistical analysis of random vectors containing different types of component variables (continuous, discrete, or categorical). Most of the common techniques to assess the relationship between random variables assume that such variables are of the same type: for example, classical Pearson’s and G-test [19,20] can be used to assess the independence between discrete variables, whereas the relationship between continuous variables has been tested using binning techniques [21], mutual information estimators [22,23,24,25], kernel-based methods [26,27], correlation distance estimators [28] or detectors based on the analysis of subsequences [29].

On the other hand, the relationship between heterogeneous variables can be evaluated, for instance, if a previous binning step is performed on the continuous variables in order to convert all of them into a discrete format; then, some of the above-mentioned procedures can be applied. In addition, note that ANOVA or MANOVA-type tests can also be employed when the dependent variables are continuous and the independent ones are categorical; unfortunately, these tests rely on very strong assumptions on the distribution of the continuous involved variables (i.e., they have to be jointly Gaussian). Recently, some tools have been developed which directly estimate the mutual information between discrete and continuous variables [30]. Most of these techniques perform well for specific settings, whereas their behavior assessment in other frameworks such as the one addressed in this paper remains a challenging problem.

Grounded on the above motivations and challenges, some algorithms to evaluate the relationship between the geolocation variables and a categorical variable are presented in this paper: we elaborate a detailed characterization and a comparative analysis of these algorithms for evaluating such relationship, making special emphasis on a new scheme based on a Voronoi tessellation (a preliminary version of this scheme was just proposed in [31] without a rigorous assessment of its capabilities). In addition, this paper illustrates the application of these algorithms to assess the geographical distribution of the communities detected in communications networks derived from CDRs. Precisely, they will allow us to shed some light on a hypothesis proposed in [18] about the distribution of communities being unrelated to geolocation within three different European cities.

The paper is organized as follows: the problem statement is formalized in Section 2, and the different existing alternatives for testing independence are presented in Section 3, including the index based on a Voronoi tessellation. All the presented alternative indexes are computationally evaluated on different scenarios in Section 4. Finally, a discussion of the results is presented in Section 5 and concluding remarks are outlined in Section 6.

## 2. Formal Problem Statement

The available data can be formally represented by a set of measurements $\left(x_{i},y_{i}\right)$, i=1,…,N, such that each $x_{i}=\left(x_{i1},x_{i2}\right)\in R^{2}$ gathers geolocation information of individual *i*, and each $y_{i}\in C=\left\{c_{1},\ldots ,c_{K}\right\}$ is a sample from a (not necessarily ordered) categorical random variable which represents some property or feature associated with such individual *i*. Accordingly, we can define $X=\left(X_{1},X_{2}\right)\in R^{2}$ to be the vector random variable determining geolocation, and Y∈C the categorical random variable which represents the mentioned property or feature. Then, each $\left(x_{i},y_{i}\right)$, which reflects an individual satisfying property $y_{i}$ at location $x_{i}$, can be seen as a sample of the joint random variables (X,Y).

In general, together with the sample set $\left(x_{i},y_{i}\right)$, i=1,…,N, the proximity between any two elements $x_{i},x_{j}\in R^{2}$ must be defined. Usually, this is formalized via a matrix *W* of weights that may generalize the usual notion of the inverse of the distance $d(x_{i},x_{j})$:

(1) $$\begin{array}{cc} w_{ij} & =\frac{1}{d(x_{i},x_{j})},\;\forall i,j\in \left\{1,\ldots ,N\right\},\;i\neq j, \end{array}$$

(2) $$\begin{array}{cc} w_{ii} & =0,\;\forall i=1,\ldots ,N. \end{array}$$

Please note that although we are focusing on geolocated data (i.e., in $R^{2}$) the reasoning and the algorithms can be extended to cases where $x_{i}\in X$ being (X,d) a metric space with a standard distance $d:X\times X\to R^{+}$.

Our main objective is to evaluate, based on the available sample, the possible relationship (i.e., dependence) between the geolocation vector variable X and the categorical variable *Y*.

In the following Section we present several tools aimed to assess such relationship.

## 3. Assessing the Relationship between X and *Y*

The most natural procedure to assess the relationship between X and *Y* is to construct a test for independence between such variables. As advanced in Section 1, usual tests for checking the independence between random variables are defined for either categorical variables [19,20] or continuous random variables or vectors [21,22,23,24,26,27,28,29]. As mentioned above, ANOVA or MANOVA-type tests [32,33] are available for cases where the dependent variables are continuous and the independent ones are categorical, but they require the distribution of the continuous variables to be jointly Gaussian (which is not usually the case for the geolocation variables).

Ripley’s K index [9], as a measure of dispersion homogeneity can be employed by analyzing the dispersion relationship between pairs of categories. This could provide an indirect way to assess independence which is not straightforward since it would involve the analysis of all possible pairs of categorical values. An extension of such index [34], based on multiple co-occurrences, could also been adapted to provide an indirect way to test for independence.

Tests of independence between a dependent categorical variable and independent continuous variables are well known for the two categories (i.e., binary) case. Nevertheless, no tests seem to be established when we have dependent categorical variables taking more than two values and continuous independent vector variables, which is the case considered in this paper.

There are two simple indirect ways to address this problem which go through transforming some variable into a new type one for allowing the application of some known test scheme. The first approach converts *Y* into a numerical variable by just assigning a different real number to each possible category. From there on, classical correlation schemes can be applied as shown below. Please note that this procedure may be quite sensitive to the arbitrary number assignment to each class. Alternatively, the second approach converts the continuous independent variables into categorical ones via a binning procedure, and then applies existing tests between categorical variables. Note also that this binning procedure may lose much information, especially when the independent variable X is a vector. A procedure based on a k-nearest neighbors analysis to estimate the mutual information has been also proposed for the scalar *X* case [30], but its extension to the case when X is a vector has not been evaluated.

In the following subsection we illustrate the first approach of transforming *Y* into a real variable, and then we show some known indexes that estimate spatial autocorrelation for real labeled data in $X\in R^{2}$.

### 3.1. Transforming *Y* into a Real Variable *Z*. Spatial Autocorrelation

Let $y\in C=\{c_{1},\ldots c_{K}\}$ so that *Y* can only take one of these *K* categorical values. Then, random variable *Y* can be transformed into a real valued random variable *Z* by defining an injective function

$$\begin{array}{cc} z:\; & C⟶R\\ & c_{i}⟶z_{i}=z\left(c_{i}\right),\;i=1,\ldots ,K \end{array}$$

which assigns a real value $z_{i}\in R$ to each categorical value $c_{i}\in C$.

Then, different indexes can be computed for the resulting Z(Y) variable with respect to X. We present now two well-known autocorrelation measures.

#### Spatial Autocorrelation

The estimation of spatial autocorrelation was addressed in [35] based on the work of [7,8]. Since then, many improvements have been proposed [36], where always z∈R, meaning that it is quantified via a numerical value. Many measurements of spatial correlation that can be defined; among them, the most common are the following ones (where we name $\bar{z}=\frac{1}{N}\sum_{i=1}^{N}z_{i}$ and $W=\sum_{i=1}^{N}\sum_{j=1}^{N}w_{ij}$).

Moran’s I

(3) $$\begin{array}{cc} I & =\frac{N}{W}\cdot \frac{\sum_{i=1}^{N}\sum_{j=1}^{N}w_{ij}\left(z_{i}-\bar{z}\right)\left(z_{j}-\bar{z}\right)}{\sum_{i=1}^{N}{\left(z_{i}-\bar{z}\right)}^{2}} \end{array}$$

Geary’s C

(4) $$\begin{array}{cc} C & =\frac{N-1}{2W}\cdot \frac{\sum_{i=1}^{N}\sum_{j=1}^{N}w_{ij}{\left(z_{i}-z_{j}\right)}^{2}}{\sum_{i=1}^{N}{\left(z_{i}-\bar{z}\right)}^{2}} \end{array}$$

Please note that these indexes provide an estimator of space autocorrelation; hence, we can formulate a test for the existence of such correlation by estimating the *p*-value corresponding to such indexes via a randomization procedure based on a random shuffling of the $z_{i}$ values while preserving the proximity matrix values $w_{ij}$. This randomization procedure will be generally performed to evaluate the relevance of the index values provided by all the algorithms proposed in this paper.

Coming back to assessing the relationship between X and *Y*, two problems arise. First, the resulting index values I and C may strongly depend on the selected assignment function *z*. Hence, some computationally demanding schemes may be required for checking different assignment functions *z* to search for the maximum index value attainable with this approach. The second limitation of these procedures is that they check for correlation, not for independence, so that they are quite sensitive to distances between points rather than focusing on their relative geolocations.

We now illustrate the above-mentioned second indirect approach to evaluate the relationship between X and *Y*.

### 3.2. Quantizing X. Mutual Information Based Index

We now illustrate the alternative procedure where the continuous variable $X\in R^{2}$ can be binned or quantized. Let us consider a partition of the region of interest into a collection of disjoint subsets $A_{l}\subset R^{2},\;l=1,\ldots ,L$, so that each subset $A_{l}$ is assigned a label category $q_{l}\in Q=\left\{q_{1},\ldots ,q_{L}\right\}$. This partition can be formulated via the function

$$\begin{array}{cc} q: & \;\;\;\;R^{2}\overset{\;}{\to }Q\\ & \left(x_{1},x_{2}\right)\overset{\;}{\to }q\left(x_{1},x_{2}\right)=q_{l},\;\;\forall \left(x_{1},x_{2}\right)\in A_{l} \end{array}$$

which assigns label $q_{l}$ to all points belonging to region $A_{l}\subset R^{2}$. Then, standard independence tests between categorical variables Q(X) and *Y* can be applied. In order to illustrate these procedures, a simple Mutual Information (MI)-based index can be computed as:

$$\begin{array}{c} M=I\left(Q,Y\right)=\sum_{q_{l}\in Q}\sum_{c_{k}\in C}p\left(q_{l},c_{k}\right)\log\left(\frac{p(q_{l},c_{k})}{p\left(q_{l}\right)p\left(c_{k}\right)}\right) \end{array}$$

Again, this alternative index can be quite sensitive to the selected quantizing function *q*. Therefore, new alternative statistics which avoid the transformation or quantization of variables may be valuable for testing the independence between X and *Y*. In the following, we present a statistic proposed in [18] which applies directly to the original X and *Y* variables.

### 3.3. Herrera’s Index

In [18] Herrera analyzed the geographical distribution of the communities detected in communications networks derived from CDRs for three different countries (France, Portugal, and Spain). The analysis was performed at both country and city scales (Paris, Lisbon and Madrid).

For assessing the relationship between geolocation and communities, a new *D* index was computed which employs the information gathered in the categories or classes (i.e., communities) $c_{k}\in C^{\prime }\subset C$ with more than one element, i.e., such that $N_{k}=\#\left\{i\in \left\{i,\ldots ,N\right\}:y_{i}=c_{k}\right\}>1$. If we define an ordering among the measurements of each class $c_{k}\in C^{\prime }$ (e.g., the ordering induced by the labeling of the whole set of measurements), and denoting $k_{i}$ the (absolute) index in the whole set for the *i*-th element of class *k*, Herrera’s index calculates:

(5) D=∑ck∈C′∑i=2Nk∑j=1i−1d(xki,xkj)∑ck∈C′∑i=2Nk(i−1)=∑ck∈C′∑i=2Nk∑j=1i−1d(xki,xkj)∑ck∈C′Nk2

Please note that this index adds up distances between pairs of elements belonging to the same class; the denominator is just a normalizing factor so that the index provides an average distance between pairs of points in the same class.

To have a baseline reference, Herrera proposed in [18] to compute also this index for a random shuffling of labels (preserving the $N_{k}$ values) on the same $x_{i}$ values; the ratio between this random-based index $D_{r}$ and the one obtained in (5) was then provided as a final indicator (Please note that for obtaining the ratio $\frac{D_{r}}{D}$ it is not necessary to compute the (same) normalizing denominator (it would cancel when computing the quotient)). This final indicator value was interpreted in [18] by saying that if the ratio was clearly large than 1, it meant that X and *Y* were not independent.

Interestingly, Herrera’s index is strongly related to a diversity index proposed in [37] where two types of distances are computed for each class. On the one hand, the average distance between the elements within each class *k* is computed an denoted as the intra-distance $d_{k}^{int}$; on the other hand, the average distance between elements of each class *k* and the elements of other classes is computed and denoted as the extra-distance $d_{k}^{ext}$:

(6) $$\begin{array}{cc} d_{k}^{int} & =\frac{\sum_{i=1}^{N_{k}}\sum_{j=1,j\neq i}^{N_{k}}d\left(x_{k_{i}},x_{k_{j}}\right)}{N_{k}\left(N_{k}-1\right)},\;\mathrm{for}\;N_{k}>1;\;\;\;d_{k}^{ext}=\frac{\sum_{i=1}^{N_{k}}\sum_{j=1}^{N-N_{k}}d\left(x_{k_{i}},x_{k_{j}}\right)}{N_{k}\left(N-N_{k}\right)},\;\mathrm{for}\;N>N_{k}. \end{array}$$

Please note that $d_{k}^{int}$ values correspond to the terms added up in the numerator of index *D*, whereas $d_{k}^{ext}$ values serve as a comparative reference. The comparison of both distances provides similar information as the one obtained by comparing intra-distances of the original data and the randomized data proposed in [18], in the sense that a data set with randomized label distribution should provide similar values for $d_{k}^{int}$ and $d_{k}^{ext}$.

The analysis in [18] using index *D* concluded that when considering country/region scales, $\frac{D_{r}}{D}$ would take values from 3.5 to 4.4, suggesting a very strong space correlation among the different detected communities. On the other hand, when the analysis was performed at a city scale (e.g., Paris, Portugal and Madrid) the $\frac{D_{r}}{D}$ index would take values between 1.08 and 1.39, much lower than the one obtained for the country scale case; based on these numbers, independence between communities and geolocation was hypothesized.

In Section 4 this procedure will be formalized by estimating the *p*-value corresponding to *D* via an appropriate randomization procedure. Please note that the same *p*-value estimation procedure could be developed by using the distances proposed in [37]. The results in the example will show that there still exists a clear dependence between communities and geolocation even at the city scale.

In the following subsection we present another index which can also be directly applied to X and *Y* variables.

### 3.4. Voronoi Tessellation Based Index

**Figure 1:**
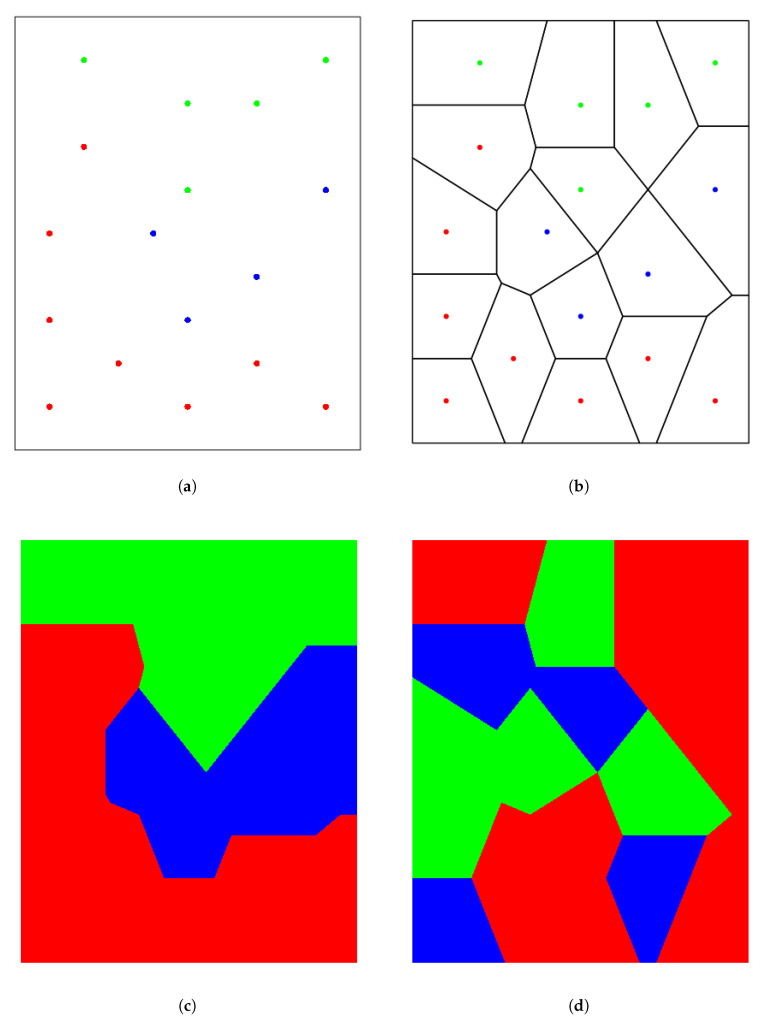
(**a**) Distribution of labeled points. (**b**) Corresponding Voronoi tessellation. (**c**) Colored groups of Voronoi cells. (**d**) Colored groups after randomly shuffling color labels.

This new index is based on the topological properties of the Voronoi tessellation [31,38] associated with the sample set $\left\{x_{1}\ldots ,x_{N}\right\}$ (with $x_{i}\in R^{2}$). This tessellation defines a partition of the space region under analysis into a collection of disjoint sub-regions (called cells) each one associated with a point $x_{i}$ (see Figure 1a,b in the example explained below). We will denote cell $V_{i}$ the one associated with $x_{i}$. Two cells in the tessellation are called adjoining if they share a common side; note that Voronoi cells $V_{i}$ and $V_{j}$ corresponding to close points $x_{i}$ and $x_{j}$ are likely to be adjoining cells. Please note that $V_{i}$ can be assigned the categorical value $y_{i}$ corresponding to $x_{i}$. Then, if adjoining cells with the same $y_{i}$ value are assembled into a single piece, the number of pieces associated with each different $y_{i}$ value in the tessellation gives information about the relationship between X and *Y*.

It is worth mentioning that sometimes we may get some Voronoi cells which happen to share a side outside the region under analysis, especially if they correspond to $x_{i}$ values located close to the boundary of such region. In order to deal with such cases in a flexible way, an extension of the algorithm proposed in [31] has been developed in this paper by incorporating an adjustable margin in the region of analysis, so that we may allow Voronoi cells to become adjoining out of the initial boundaries of the original region of analysis.

Figure 1 illustrates the information provided by the Voronoi tessellation procedure in a simple example. Figure 1a displays a distribution of points where the label *y* can take three values represented by three colors (red, blue, and green). The corresponding Voronoi tessellation is shown in Figure 1b whereas the cells are colored according to their corresponding *y* value in Figure 1c. Please note that only one piece is obtained for each color due to the very strong relationship between X and *Y*. Finally, Figure 1d shows the result after shuffling the label values among the points: a larger number of pieces is obtained for independent X and *Y*.

To efficiently perform the computation of the number of pieces (groups of adjoining cells) associated with each label $y_{i}$, note that the Voronoi tessellation adjacency structure can be modelled via a graph *G* where each vertex represents a cell $V_{i}$ (with label $y_{i}$), and two vertices are connected if their corresponding cells are adjoining. By selecting only those nodes with a given label value $y_{i}=c_{k}$ the corresponding subgraph $G_{k}$ (composed only by the selected nodes and the corresponding links) gathers all the required information for determining the pieces or groups of cells. Precisely, for each class *k* we can easily determine both the corresponding number of pieces and the size (number of cells) of each piece by computing the connected components $CC_{k}^{l}$ of the corresponding subgraph $G_{k}$ (note that each $CC_{k}^{l},\;l=1,\ldots ,L_{k}$ is again a connected subgraph of $G_{k}$). We can denote $|CC_{k}^{l}|$ the number of nodes of each connected component $CC_{k}^{l}$. Please note that $\sum_{l=1}^{L_{k}}\left|CC_{k}^{l}\right|=N_{k}$, the number of nodes of subgraph $G_{k}$.

Once the number of pieces associated with each class are computed, an entropy measure is proposed to comparatively quantify the distribution of such number of pieces among all classes. In the same way as Herrera’s index, the Voronoi index only gathers the information in classes $c_{k}\in C^{\prime \prime }\subset C$ which have two or more elements, i.e., satisfying $N_{k}=\#\left\{i\in \left\{i,\ldots ,N\right\}:y_{i}=c_{k}\right\}\geq 1$. For each class the index computes the ratio between the entropy of the distribution of the sizes of the corresponding connected components and the maximal entropy associated with the overall size (total number of cells) of such class. Finally, the index is obtained by adding up these entropy ratios:

(7) $$\begin{array}{cc} E & =-\sum_{c_{k}\in C^{\prime \prime }}\frac{\sum_{l=1}^{L_{k}}\frac{|CC_{k}^{l}|}{N_{k}}\log\left(\frac{|CC_{k}^{l}|}{N_{k}}\right)}{\log(N_{k})} \end{array}$$

Please note that this index gathers information on the relative distribution of the labels on the points, so that it is not affected by distance scaling transformations. Finally, the use and interpretation of this new index can be formalized, once again, by estimating the *p*-value associated with the obtained value of *E* via an appropriate randomization simulation scheme.

## 4. Comparative Evaluation of Indexes

### 4.1. Simulation Examples

The following family of examples comparatively illustrates how the performance of the different indexes depends on the size and shape of the geographical regions associated with the different categories. We consider a region $R=\left[-5,5\right]\times \left[-1,1\right]\subset R^{2}$ where we can define three sub-regions whose size, range and shape depend on the value of a real parameter a∈[0,5]:

$$\begin{array}{cc} R_{1}\left(a\right) & =\{\left(x_{1},x_{2}\right)\in R||x_{2}-\sin\left(x_{1}\right)|<0.25\;\mathrm{and}\;|x_{1}|<a\},\\ R_{2}\left(a\right) & =\{\left(x_{1},x_{2}\right)\in R|\left(x_{1},x_{2}\right)\in R\backslash R_{1}\left(a\right)\;\mathrm{and}\;|x_{1}+x_{2}|<0.25\},\\ R_{3}\left(a\right) & =R\backslash (R_{1}\left(a\right)\cup R_{2}\left(a\right)). \end{array}$$

By selecting the value for a∈[0,5] we can adjust the size of $R_{1}\left(a\right)$ which has the shape of a merging (thick) sine graph in R; this variation will allow a characterization of the sensitivity of the indexes to the size and shape of the regions. Now, each point x is assigned a label value y(x)∈{Red,Blue,Green} according to the following distribution:

(8) $$\begin{array}{c} \begin{array}{cc} P(Y\;=\;R)=0.9,P(Y\;=\;B)=P(Y\;=\;G)=0.05, & \mathrm{if}\;\left(x_{1},x_{2}\right)\in R_{1}\left(a\right),\\ P(Y\;=\;B)=0.9,P(Y\;=\;R)=P(Y\;=\;G)=0.05, & \mathrm{if}\;\left(x_{1},x_{2}\right)\in R_{2}\left(a\right),\\ P\left(Y\;=\;R\right)=P\left(Y\;=\;B\right)=P\left(Y\;=\;G\right)=\frac{1}{3}, & \mathrm{if}\;\left(x_{1},x_{2}\right)\in R_{3}\left(a\right). \end{array} \end{array}$$

Based on taking uniform samples in R following such distribution, we now analyze the performance of the different indexes.

#### 4.1.1. Detailed Analysis for a=2.5

**Figure 2:**
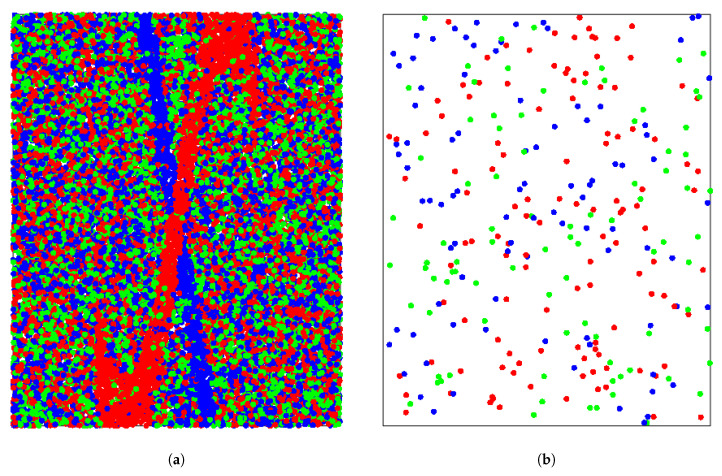

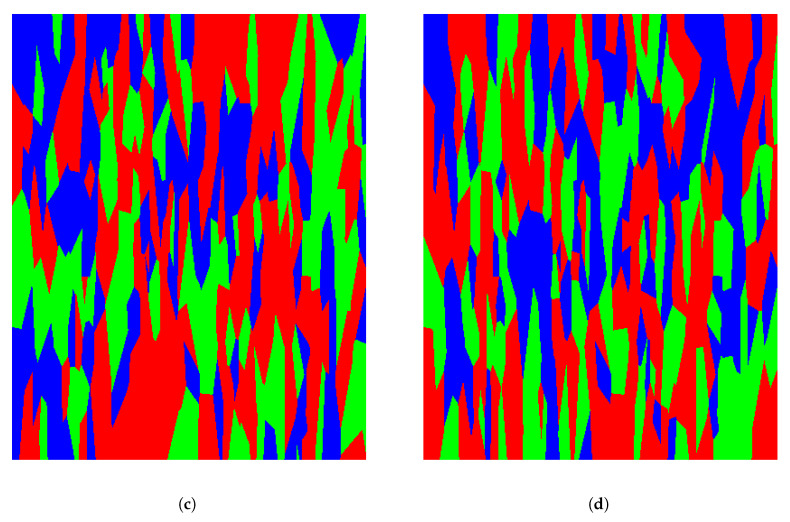
(**a**) Distribution of 25,000 sampled labeled points. (**b**) Distribution of 500 sampled labeled points. (**c**) Colored groups of Voronoi cells for sample of 500 points. (**d**) Colored groups after randomly shuffling color labels for sample of 300 points.

For the case of a=2.5, Figure 2a shows the spatial distribution of a sample (X following a uniform distribution within R) of 25000 points, which have been color labeled according to the y(x) distribution in (8); the only purpose of this large number of points is to clearly delineate the regions $R_{i}\left(2.5\right),\;i=1,2,3$. Figure 2b displays the spatial distribution of only the first 300 sampled points and, for this case, Figure 2c shows the colored groups of the corresponding Voronoi cells. Finally, for comparative purposes, Figure 2d displays the Voronoi cells for the same spatially distributed 300 points after having performed a random shuffling of the color labels.

Monte Carlo simulations were performed by taking different samples of size 300 to estimate the *p*-values corresponding to the Moran’s I, Geary’s C, Mutual Information M, Herrera’s ratio D and the Voronoi-based index E. Since, as mentioned above, Moran’s I and Geary’s are sensitive to the number assignment function *z*, we assigned numbers to the labels in two different ways, $z_{1}$ and $z_{2}$, so that: $z_{1}\left(R\right)=0$, $z_{1}\left(B\right)=1$, $z_{1}\left(G\right)=2$ and $z_{2}\left(R\right)=0$, $z_{2}\left(B\right)=2$, $z_{1}\left(G\right)=1$.

For each sample of size 300 a randomization technique was applied 200 times, by randomly shuffling the labels among all data geolocations, for estimating the *p*-value corresponding to each index. 200 Monte Carlo simulations were performed to assess the distribution of the estimated *p*-values. Table 1 shows a summary of the *p*-value distribution for the different indexes when applied to the data generated in this example.

Please note that the Voronoi index provides the smallest expected *p*-value with also smallest standard deviation. The rest of indexes display similar behaviors.

#### 4.1.2. Influence of the Value of Parameter *a*

**Figure 3:**
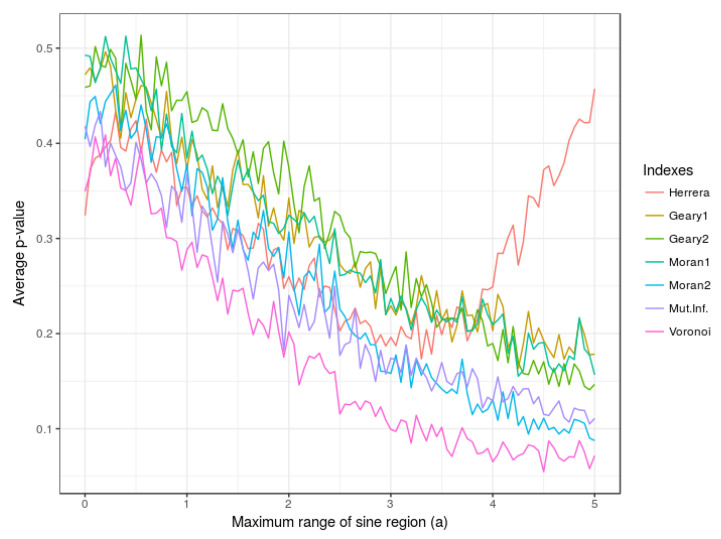
Variation of average *p*-values as a function of *a* corresponding to Moran’s I, Geary’s C, Mutual Information M, Herrera’s D and the Voronoi-based index E. Number of points = 300; randomization iterations = 200; Monte Carlo iterations = 200.

In this subsection, the performance of the indexes is evaluated for different sizes and forms of region $R_{1}\left(a\right)$ whose scope can be regulated as a function of the parameter *a*. In Figure 3 the expected *p*-value associated with each one of the indexes is displayed as a function of such parameter *a*. Note again that the Voronoi index consistently provides the smallest expected *p*-value. The rest of indexes perform well except for Herrera’s index D which seems to have trouble for capturing the relationship when region $R_{1}\left(a\right)$ has a large range (i.e., the parameter *a* takes values close to 5).

### 4.2. Telephone Social Network Community Distribution in Cities

In [18] three social networks (in Portugal, Spain and France) were constructed derived from the communication activity between phone users, based on the CDRs registered by a telephone operator in those three countries. Then, communities were computed in those networks by applying the Louvain algorithm [17]. Next, also in [18], the geographical distribution of those communities along the telephone towers was computed by assigning to each tower the prevailing social network community among the phone users geographically associated with such tower. Finally, the relationship between communities and their geolocations was analyzed at country and city scales (Lisbon, Madrid, and Paris). The dependency of the communities on the geolocation was so high at country scale when compared to the city scale that an independence hypothesis was formulated at the city scale, claiming that the community distribution in cities is not affected by geolocation. We show now that the indexes presented in this work allow us to shed some light on this hypothesis. Table 2 shows the estimated *p*-values of the different indexes when applied to the data provided in [18] corresponding to the geographical distribution of communities along the telephone towers of the three cities (only best results between $z_{1}$ and $z_{2}$ are displayed for Moran’s I and Geary’s C). Please note that the extremely small *p*-values have been estimated by assuming an approximate Gaussian distribution of the corresponding index *p*-value and estimating its standard deviation via, again, a randomization procedure.

All indexes (except for Moran’s I and Geary’s C in Lisbon) suggest a strong dependency of communities on geolocation for all the cities. It is worth mentioning that Herrera’s index, provided that a rigorous *p*-value analysis is performed, does also detect such dependency, even though it is weaker than the dependencies detected at the country scales. Hence, it was the lack of a *p*-value analysis in [18], where only absolute values of the index where relatively compared at both scales, that lead to a misleading independence hypothesis at the city scale.

**Figure 4:**
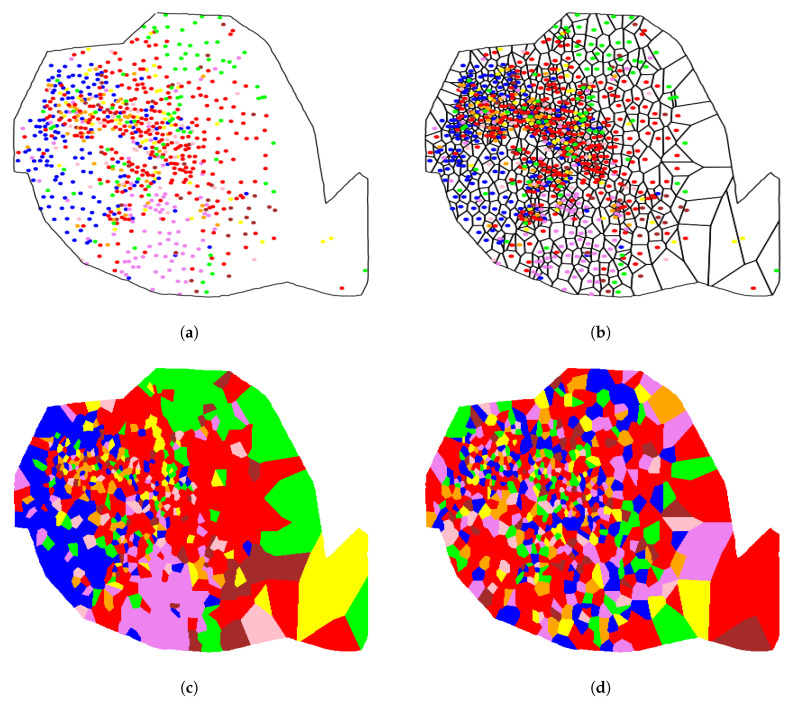
Analysis of Paris: (**a**) Community distribution along the telephone towers. (**b**) Corresponding Voronoi tessellation. (**c**) Colored groups of Voronoi cells. (**d**) Colored groups after randomly shuffling color labels.

For illustrative purposes, the community distribution along the towers for Paris is shown in Figure 4a, where each point presents a tower location and the corresponding color indicates the prevailing social network community in such tower. Figure 4b represents the corresponding Voronoi tessellation and Figure 4c displays the cells colored according to their corresponding *y* value. Finally, Figure 4d shows the respective results after shuffling the label values among the points, for comparative purposes. Visual inspection suggests that the number of pieces is clearly larger in the randomly shuffled case, this fact being in accordance with the extremely *p*-value obtained in Table 2.

## 5. Discussion

The ability of the presented indexes to evaluate the role of geolocation in the categorical variable depends on the size and shape of regions where the categories may be distributed. The new index based on the Voronoi tessellation, besides being directly applicable, has shown also a quite robust and efficient performance (with computational complexity nlogn) when compared with other indexes. It would be of interest to study further the topological properties of this new index, specifically the invariance against deformations beyond the group of motions in the plane. These techniques can be applied in many real scenarios as the one illustrated with the distribution of communities in cities.

## 6. Conclusions

Several indexes have been evaluated for assessing the relationship between geolocation (latitude and longitude) and a categorical variable, given a corresponding sample set. The direct application of the new index based on a Voronoi tessellation has proven to be robust when applied to different examples, including the geolocation of communities in some communication networks derived from CDRs. 

## Figures and Tables

**Table 1:** Summary of distribution of estimated *p*-values corresponding to Moran’s I, Geary’s C, Mutual Information M, Herrera’s ratio D and the Voronoi-based index E. Number of points = 300; randomization iterations = 200; Monte Carlo iterations = 200.

	Min	1st Q.	Median	3rd Q.	Max	Mean	Std
Moran I ($z_{1}$)	0	0.03	0.135	0.37	0.98	0.237	0.262242
Moran I ($z_{2}$)	0	0.01	0.07	0.23	0.99	0.18735	0.261614
Geary C ($z_{1}$)	0	0.02	0.13	0.3925	0.97	0.2405	0.267861
Geary C ($z_{2}$)	0	0.03	0.15	0.43	0.99	0.2764	0.292252
Mut. Inf. M	0	0.02	0.135	0.305	0.975	0.207925	0.237545
Herrera D	0	0.05375	0.1835	0.3825	0.935	0.239050	0.224115
Voronoi E	0	0.0150	0.06	0.18	0.815	0.128475	0.172679

**Table 2:** Estimation of *p*-values corresponding to the different indexes when testing the distribution of communities in Lisbon, Madrid and Paris.

	Paris	Lisbon	Madrid
Moran I	$10^{-124}$	0.34	0.002
Geary C	$10^{-18}$	0.37	0.0016
Mut. Inf. M	$10^{-82}$	0.0007	0.046
Herrera D	$10^{-36}$	0.0036	0.000034
Voronoi E	$10^{-111}$	$10^{-15}$	0.0045

## Abbreviations

The following abbreviations are used in this manuscript:

CDRs	Call Detail Records
ANOVA	Analysis of variance
MANOVA	Multivariate Analysis of Variance
